# A study on the relationship and path between mental health and burnout of Chinese athletes

**DOI:** 10.3389/fpsyg.2024.1422207

**Published:** 2024-07-19

**Authors:** Yun Gao, Lei Wang

**Affiliations:** ^1^Department of Physical Education, Shanghai Jiao Tong University, Shanghai, China; ^2^School of Physical Education, Shanghai University of Sport, Shanghai, China

**Keywords:** competitive athletes, mental health, athlete burnout, influence path, COVID-19

## Abstract

**Background:**

This study aims to explore the relationship and influencing pathways between mental health indicators and athlete burnout among Chinese competitive athletes.

**Methods:**

A cross-sectional study was conducted on 501 elite Chinese athletes from several national and provincial sports teams. Generalized Anxiety Disorder Scale (GAD-7) was used to measure anxiety, Patient Health Questionnaire (PHQ- 9) was used to measure depression, Athlete Psychological Strain Questionnaire (APSQ) was used to measure perceived stress, and Athlete Burnout Questionnaire (ABQ) was used to measure burnout. The results were derived using reliability testing, descriptive statistics, correlation analyses, and structural equation modeling.

**Results:**

The following results were obtained: (a) the clinical detection rates of anxiety and depression in this sample were within normal levels, but the detection rate of perceived stress (78.64%) was relatively high; (b) all three mental health indicators were significantly correlated with athlete burnout; (c) in our model, there was a direct path with APSQ directly contributing to 69.95% of the variance in athlete burnout, and two indirect paths with APSQ exerting an indirect effect through depression or/and anxiety accounting for 30.05% of the variance.

**Conclusion:**

The findings revealed the psychological characteristics of Chinese competitive athletes and the direct and indirect effects of the APSQ on burnout. Future research should actively promote the international development and application of burnout assessment tools, conduct more comprehensive studies on athlete mental health monitoring, and intensify efforts in athlete education, treatment, and support services, as well as strategies for athlete’s coping stress.

## 1 Introduction

### 1.1 Mental health issues in competitive athletes

China’s competitive sports have achieved remarkable success and garnered international recognition. According to statistics from international sports organizations, Chinese athletes wins medals in major international competitions such as the Olympics, World Cups, and World Championships, solidifying its strength and influence in competitive sports (IOC, 2022). These outstanding achievements are closely linked to the well-established training system (Xu et al., 2019). The cultivation of young sports talents, comprehensive management and support, and financial investments have also laid a solid foundation for the rapid development of China’s sports industry (Zhou and Xiao, 2023).

However, alongside these outstanding competitive performances, the mental health of athletes has become a growing concern. Heavy training loads and competitive pressures can lead to anxiety, depression, and other psychological issues. The COVID-19 pandemic has further exacerbated these psychological pressures on athletes and has become a focal point of international attention (Liu S. et al., 2023; Liu Y. et al., 2023). Research indicates that psychological adaptability, self-esteem, and self-efficacy issues can significantly impact athletic performance (Huang and Zhong, 2021), training motivation, and even their career paths (Reardon, 2023).

Addressing the mental health concerns of high-level athletes, developing psychological health education programs, and providing psychological counseling services are imperative in preparing them for the rigors of training and competitions. In 2020, the General Administration of Sport of China introduced the “Psychological Handbook for Active Athletes, “and in the “14th Five-Year Plan for Sports Development” published in 2021, it emphasized the need to promote the application of psychological training for athletes to take competitive sports to a new level (General Administration of Sport of China, 2021a,b). In March 2019, the International Olympic Committee (IOC) released a consensus statement on “Mental Health in Elite Athletes” (Reardon et al., 2019), proposing standardized evidence-based assessment methods for mental health symptoms among elite athletes. In the same year, the International Society of Sports Psychology (ISSP) also issued a joint statement on “Improving Mental Health for High Performance Athletes” (Henriksen et al., 2019), calling for the definition of context-specific mental health in sports and the development of comprehensive psychological assessment strategies.

Common mental health issues among athletes include anxiety, depression, and stress. Anxiety is the most prevalent psychological symptom among athletes. It is characterized by physical and psychological responses when individuals face threats or pressure, with symptoms such as tension, restlessness, fear, anxiety, increased heart rate, rapid breathing, muscle tension, headaches, insomnia, fatigue, and difficulty concentrating (American Psychiatric Association, 2013). Anxiety can be a transient state dependent on a situation or event (state anxiety) or a relatively stable personality trait (trait anxiety) (Rice et al., 2019a). Tools such as the Sport Anxiety Scale (SAS) (Smith and Smoll, 1990) and the Generalized Anxiety Disorder scale (GAD-7) (Spitzer et al., 2006) are used to measure anxiety levels. Factors influencing anxiety include individual, environmental (Rocha et al., 2018), sports-related (Correia and Rosado, 2019) and lifestyle factors (Ozen et al., 2020; Gao et al., 2021). In addition to anxiety, depression is also relatively common among athletes. Depression is characterized by mood changes, loss of interest or pleasure in daily activities, along with sleep and eating problems, lack of energy, difficulty concentrating, and feelings of low self-worth. Data indicates that depression affects 6.7% of the global adult population, and risk factors for athletes include injuries (Mainwaring et al., 2010), overtraining (Meehan et al., 2004), and decreased sports performance (Nederhof et al., 2006). Severe depression can lead to extreme behaviors in athletes (Onate, 2019). Protective factors include team support (Armstrong and Oomen-Early, 2009). Assessment tools for depression include the Beck Depression Inventory (BDI) (Beck et al., 1961), the Center for Epidemiologic Studies Depression Scale (CES-D) (Radloff, 1997), and the Patient Health Questionnaire (PHQ-9) (Kroenke et al., 2001). Athlete psychological strain refers to the various psychological challenges and pressures they experience during sports competitions. This stress perception is brought about by the unique nature of competitive sports, which involves high competitiveness, significant responsibilities, and high expectations (Endo et al., 2023). While moderate psychological strain can enhance athletic performance (Helgeson, 1994), prolonged and intense stress can negatively impact athletes’ physical and mental health (Lu et al., 2016). Tools such as the Kessler-10 (K10) (Kessler et al., 2002) and the Athlete Psychological Pressure Scale (APSQ) (Rice et al., 2019a,b) are used to measure athlete psychological strain.

Presently, the primary approaches to supporting the mental health of elite athletes involve pharmacological intervention and other alternative therapies. Pharmacological assistance is typically targeted at athletes with diagnosed psychiatric symptoms reaching clinical thresholds, but it necessitates careful consideration of specific issues, such as potential side effects and adherence to The World Anti-doping Code (Reardon and Factor, 2010). A comprehensive review by Reardon delves into the use of medication in managing athletes’ mental health (Reardon and Creado, 2016). Cognitive Behavioral Therapy (CBT) stands as a widely employed intervention, with empirical evidence demonstrating its efficacy among elite athletes (Reardon et al., 2019). Other therapies like Rational Emotive Behavior Therapy (REBT) (Turner, 2016) and Mindfulness (Birrer et al., 2012) have also found application in this context, albeit to a lesser extent.

### 1.2 Burnout in competitive athletes

Athlete burnout is defined as a cognitive-affective syndrome comprised of emotional and physical exhaustion, a reduced sense of accomplishment, and sport devaluation (Gustafsson et al., 2017). As athletes engage in long-term training and competitions, they inevitably experience certain levels of burnout. Burnout in athletes is defined as a syndrome characterized by emotional and physical exhaustion, reduced sense of accomplishment in sports, and devaluation of sports participation, leading to a withdrawal from the sports domain (Cureton, 2009). Some scholars define it as sports-related psychological fatigue (Li et al., 2006). Emotional and physical exhaustion is manifested by a perception of resource depletion due to training or competitions; reduced sense of accomplishment is reflected in a tendency to negatively evaluate one’s athletic abilities and achievements; and sports devaluation indicates a cynical attitude towards sports participation. Regarding the formation mechanism of athlete burnout, Gould pointed out that overtraining may be the primary cause of burnout (Gould et al., 1996). In addition, occupational environment is also considered to be closely related (Thomas et al., 2021).

There are three theoretical models concerning the formation of athlete burnout: (1) First is the Cognitive-Affective Stress Model, which posits that professional burnout in athletes is a stress-based process influenced by personality and motivational factors (Smith, 1986). (2) Second is the Burnout-Stress Perception Model, which suggests that stress is not a cause of burnout but rather a symptom of it (Coakley, 1992). (3) Third is the application of Self-Determination Theory to explain it (Ryan and Deci, 2000), where the frustration of psychological needs in athletes can lead to burnout (Liu S. et al., 2023; Liu Y. et al., 2023). Measurement tools for athlete burnout include the Maslach Burnout Inventory (Cresswell and Eklund, 2006), the Eades Burnout Questionnaire (Gustafsson et al., 2007), and the more recently developed Athlete Burnout Questionnaire (ABQ) (Raedeke and Smith, 2001).

Athlete burnout has been found to be intricately linked with anxiety levels (Katkat, 2015) and is associated with perceived stress (Zhang and Zhi, 2018), job satisfaction (Lee et al., 2017), and social support (Zhao et al., 2022) among athletes. Factors such as perfectionism, fear of failure, resilience, and extrinsic motivation have also been shown to significantly predict burnout in this population (Yıldırım et al., 2023a). The COVID-19 pandemic (2019-nCoV) has had profound implications on human life, with the World Health Organization (WHO) declaring it a Public Health Emergency of International Concern (PHEIC) in January 2020. The Tokyo Olympics was subsequently postponed, disrupting the normative routines and mobility of individuals, and sparking social anxiety due to the severity of the cases. Athletes, as a distinct group, face dual pressures of pandemic-related fears and training demands daily.

Research has revealed that COVID-19 has had a significant negative impact on burnout among young individuals (Yıldırım and Ashraf, 2023), university students (Chen et al., 2024), athletes (Yıldırım et al., 2023b), healthcare professionals (Chirico et al., 2023), and emergency responders, including both short-term and latent psychological trauma (Nucera et al., 2023). Studies focusing on athlete burnout can play a crucial role in preventing and mitigating the development of mental health issues, thereby averting exacerbation of these concerns (Nucera et al., 2023).

Consequently, this study aims to explore the psychological state characteristics of athletes, investigate the interrelationships between psychological strain, anxiety, depression, and burnout, as well as the dynamic pathways between mental health indicators and burnout. The primary objective is to contribute to the early identification and prevention of this issue. Hypothesis 1 posits a significant positive correlation between the athlete psychological strain, anxiety, and depression with burnout. Hypothesis 2 proposes that athlete psychological strain, anxiety, and depression exert an influence on burnout, with one or more of these variables potentially acting as mediating factors.

## 2 Research methods

### 2.1 Participants

The target population for this study is active athletes who are at least 18 years old with grades ranging from Division I Athlete, Master Athlete, to International Master Athlete, based on the standards set by the Chinese National Sports Administration (General Administration of Sport of China, 2021a,b). The standards for each sport’s athlete classification may differ. This research employed an online survey tool, “Sojump” (WJX.cn), to design the questionnaire. The survey distribution was carried out through non-official channels, leveraging the personal connections between coaches and athletes, using a snowball sampling method. Athletes provided informed consent online before receiving the survey link, ensuring anonymity in data submission.

Data exclusion criteria included: (1) under 18 years old; (2) not meeting Division I standards or above; (3) non-active athletes; (4) duplicate submissions; (5) insufficient completion time (Less than 300 s).

### 2.2 Procedure

After obtaining ethical approval from the institution of the corresponding author, the survey questionnaire was initially tested on sports teams through the first and corresponding author’s affiliations. Subsequently, the study details and survey link were shared on social media and sent to key stakeholders, such as coaches from Beijing Sport University and East China Normal University, and they were asked to distribute the survey link to their student athletes. Subsequently, coaches from Shanghai and Zhejiang training at the national training centers were contacted, who were instructed to share the link with other coaches, who in turn distributed it to their athletes. The questionnaire spread gradually to teams specializing in table tennis, gymnastics, track and field, boxing, shooting, synchronized swimming, tennis, and rowing. Finally, links were sent to athletes on several teams in Guangdong. In addition, student athletes from the authors’ institutions were also publicly recruited. Athletes were instructed to share the study information with anyone they knew who met the inclusion criteria. Data collection took place between October and December 2022.

Before conducting the online survey, athletes were made aware that their participation would remain anonymous, with no information shared with others such as coaches. They were informed that they could withdraw from the study at any time after completing it, and they were not obligated to answer any questions they were uncomfortable with. Only the research team would have access to their data. Following each survey completion, athletes received personalized links to view their respective scale results.

A total of 501 valid samples were confirmed, as detailed in Table 1. Among them, 413 athletes (56.6%) were at Division 1, 269 (36.8%) were Master Athletes, and 48 (6.6%) were International Master Athletes. These athletes represented 22 distinct sports, with the top five being Athletics (21.8%), Diving (14.7%), Swimming (12.2%), Gymnastics (9.9%), and Tennis (8.9%). For further detail regarding the participants’ demographics, please refer to Appendix Table A1.

**Table 1:** Demographic characteristics of the participants.

**Variable**	**Frequency**	**Percent %**
Gender		
Male	256	51.10
Female	245	48.90
Age		
18 ~ 20	285	56.89
>20	216	43.11
Athletes level		
International masters	43	8.58
Masters	201	40.12
Division I	257	51.30
Sport discipline		
Swimming	78	15.57
Diving	71	14.17
Track and field	70	13.97
Tennis	45	8.98
Acrobatics	33	6.59
Soccer	30	5.99
Table Tennis	28	5.59
Gymnastics	25	4.99
Others	121	24.15

### 2.3 Measurement

This study employed a comprehensive survey tailored for the athlete population, utilizing validated scales. The questionnaire encompassed several sections, initially gathering participants’ demographic information, such as gender, age, location, athletic level, and sport discipline. The following sections featured an overview of the selected mental health and athlete burnout assessment tools, along with adjustments made based on the data collected in this study.

The validation criteria for scale reliability and validity were as follows: (a) each individual item’s correlation with the total scale score needed to exceed 0.4 (Cronbach, 1951). (b) the sample was randomly divided into two equal subsets for analysis. The first subset was employed in an exploratory factor analysis to examine if the extracted principal factors matched the original scale’s design (Fornell and Larcker, 1981). (c) If there was a discrepancy in the primary factor structure, the dimensions were redefined based on the factor extraction results (Hair, 2014). (d) a confirmatory factor analysis was then conducted using the second subset, ensuring that the model fit conformed to established criteria (Kline, 2016). This process led to the final determination of the revised scale’s structure.

Anxiety Scale (GAD-7) This scale, with 7 items, assesses anxiety (e.g., “How often have you been bothered by feeling nervous, anxious, or on edge in the past 2 weeks?”), using a 5-point Likert scale (0 = “Not at all” to 3 = “Nearly every day”). Scores range from 0 to 21, with scores below 5 indicating no anxiety, 5–9 mild, 10–14 moderate, and 15+ severe. A score of 10 or above generally suggests clinical concern. GAD-7 has undergone extensive validity and reliability testing (Gouttebarge et al., 2021). The scale’s internal consistency α = 0.920, indicating good reliability (Yu and He, 2004).

Depression Scale (PHQ-9) This scale, with 9 items, evaluates depression (e.g., “How many days were you bothered by feeling down, depressed, or hopeless in the past 2 weeks?”), using a 5-point Likert scale. Scores range from 0 to 27, with scores below 5 indicating no depression, 5–9 mild, 10–14 moderate, 15–19 moderate to severe, and 20+ severe. A score of 10 or above typically prompts clinical attention. PHQ-9 has been widely validated for reliability and validity (Kroenke et al., 2001). The scale’s internal consistency *α* = 0.896.

Athlete Psychological Strain Questionnaire (APSQ) The APSQ scale consists of 10 items to assess athletes’ psychological strain (e.g., “It’s difficult for me to get along with my teammates,” “I find it hard to motivate myself,” etc.), using a 5-point Likert scale from 1 to 5. The total score ranges from 10 to 50, with scores below 15 indicating no stress, 15–16 moderate, 17–19 high, and 20+ very high. A score of 17 or above generally suggests clinical attention. The scale has three dimensions: Self-Control Difficulties (items 1–4), Performance Anxiety (items 5–8), and External Coping (items 9–10). It exhibits high internal consistency among male athletes (Cronbach’s *α* = 0.87) and female athletes (*α* = 0.84) (Rice et al., 2020), and is included in the International Olympic Committee’s Sport Mental Health Assessment Tool (SMHAT-1) (Anderson et al., 2023). After correlation analysis and exploratory factor analysis, items 1 and 10 were excluded. The adapted scale consists of two dimensions: Dimension 1 - “Current Self-Control” (original items 2 ~ 5), Dimension 2 - “Future Stress Coping” (original items 6 ~ 9). The new scale structure was ideally validated by confirmatory factor analysis of structural equations. The internal consistency of the scale is moderate (α = 0.756).

The Exercise Exhaustion Scale (ABQ) (Raedeke and Smith, 2001) consists of three dimensions with 15 items: Physical/Energetic Exhaustion (items 2, 4, 8, 10, 12), Reduced Sense of Achievement (items 1, 5, 7, 13, 14), and Devaluation of Exercise (items 3, 6, 9, 11, 15). Higher scores indicate greater levels of exercise exhaustion, with items 1 and 14 reverse-scored. The scale uses a 5-point Likert scale, ranging from 0 (never) to 4 (always). In samples from athletes in countries like China and the United States, the validity has shown to be satisfactory (Liu et al., 2022). Following correlation analysis and exploratory factor analysis, which led to the exclusion of the original items 1, 3, and 14, the results of the validation for the modified scale structure model were favorable. The newly adapted 12-item ABQ scale exhibited strong internal consistency (α = 0.910).

### 2.4 Data processing

In this study, data were analyzed using IBM SPSS v26 for descriptive statistics, inter-variable correlations, and exploratory factor analysis. The structural equation modeling was conducted with Amos 23.0 to assess the fit of the scales and the mediating effect of the APSQ. Maximum likelihood estimation was employed to calculate the estimates. According to Kline and Hooper’s recommendations, the adequacy criteria for a structural equation model’s fit include: Comparative Fit Index (CFI) > 0.9, Tucker-Lewis Index (TLI) > 0.9, and the Root Mean Square Error of Approximation (RMSEA) < 0.08. The ratio of Chi-square to degrees of freedom (*X*^2^/df) should be between 1 and 3, indicating acceptable fit (Hooper and Khawaja, 2010; Kline, 2016). The bias-corrected bootstrapped tests for indirect effects were employed, using 2000 resamples. The confidence intervals were calculated at the 95% level, with sample means used to estimate missing data points.

## 3 Results

### 3.1 Main indicators

### 3.2 Common method bias

Approach Due to the reliance on self-reported questionnaire data, this study employed Harman’s single-factor test to examine for common method bias (CMB) in all items of the mental health and athlete burnout scales. The unrotated exploratory factor analysis revealed six factors with eigenvalues greater than 1, with the first factor accounting for 38.6% of the variance, which is below the commonly accepted threshold of 40%. Consequently, the data in this study do not indicate significant common method bias (Tables 2, 3).

**Table 2:** The main indicators and the distribution characteristics between sex and sport level.

Indicators	All (*N* = 501)	Skew	Kurtosis	Male (*N* = 256)	Female (*N* = 245)	International masters (*N* = 43)	Masters (*N* = 201)	First-class (*N* = 257)
Age	20.74(2.51)	1.050	0.57	21.04(2.7)	20.42(2.26)	23.49(3.17)	21.28(2.54)	19.85(1.84)
GAD7	5.03(4.33)	1.090	1.60	4.68(4.46)	5.39(4.16)	5.70(5.17)	5.22(4.17)	4.77(4.29)
PHQ9	5.81(4.65)	1.190	2.36	5.50(4.94)	6.14(4.31)	5.79(5.13)	5.91(4.55)	5.74(4.67)
APSQ8	18.28(5.51)	0.250	−0.19	17.88(5.83)	18.70(5.12)	18.09(5.52)	18.69(5.58)	17.99(5.45)
ABQ12	16.73(9.26)	0.620	0.26	15.96(9.66)	17.53(8.77)	15.67(7.29)	17.00(9.89)	16.69(9.06)

GAD7, Generalized Anxiety Scale; PHQ9, Self-rating Depression Scale; APSQ8, Adapted athletes psychological strain scale; ABQ12, Adapted athlete Burnout scale.

**Table 3:** Correlation between levels of anxiety, depression, psychological strain and athlete burnout.

Indicators	GAD7	PHQ9	APSQ8			ABQ12			
				-Status quo self-control	-Future stress coping		-Physical/emotional exhaustion	-reduced sense of accomplishment	-sport devaluation
PHQ9	0.79**	1							
APSQ8	0.66**	0.66**	1						
-Status quo self-control	0.62**	0.62**	0.91**	1					
-Future stress coping	0.57**	0.58**	0.90**	0.64**	1				
ABQ12	0.58**	0.67**	0.66**	0.62**	0.58**	1			
-Physical/emotional exhaustion	0.54**	0.65**	0.63**	0.57**	0.57**	0.91**	1		
-Reduced sense of accomplishment	0.48**	0.56**	0.53**	0.55**	0.41**	0.87**	0.68**	1	
-Sport devaluation	0.45**	0.46**	0.54**	0.47**	0.50**	0.77**	0.56**	0.55**	1

Gender, athlete grade and sport discipline were taken as covariables. **p<0.01.

### 3.3 Correlation between mental health and athlete burnout

In this study, the scores of the new athlete burnout scale ABQ-12 and its 3 sub-dimensions were significantly positively correlated with the total scores of anxiety, depression and APSQ-8 and its 2 sub-dimensions.

### 3.4 Path analysis of the impact between psychological indicators and athlete burnout

To investigate the path between psychological indicators and athlete burnout, a confirmatory factor analysis (CFA) was conducted using 21 observed variables (7 subscales of anxiety [GAD], 9 subscales of depression [PHQ], 2 subscales of psychological pressure [APSQ], and 3 subscales of burnout [ABQ]). The ML method was employed, with the results showing *X*^2^ = 673.74, df = 183, *X*^2^/df = 3.68, *p* = 0.000, TLI = 0.91, GFI = 0.88, CFI = 0.92, RMSEA = 0.07, and SRMR = 0.04. The model’s fit was considered acceptable, with significant and well-standardized path coefficients between the latent variables. After refining the internal relationships among subscales based on the modification indices (Jöreskog and Sörbom, 1993), the model was re-fitted, resulting in improved fit (*X*^2^ = 395.62, df = 167, *X*^2^/df = 2.37, *p* < 0.001, GFI = 0.93, CFI = 0.96, RMSEA = 0.05, SRMR = 0.03) (Table 4).

**Table 4:** Reliability and mean variance extraction of measurement model.

Indicator	CR	AVE	Correlation coefficient(Φ)		
			Anxiety	Depression	Psychological strain
Anxiety	0.91	0.58	1		
Depression	0.87	0.43	0.91(0.35)	1	
Psychological strain	0.77	0.62	0.78(0.99)	0.82(0.78)	1
Athlete burnout	0.81	0.59	0.67(1.11)	0.80(0.93)	0.83(17.33)

The model’s structural reliability values for all indicators range from 0.76 to 0.91, all exceeding 0.7, which meets the criteria (Fornell and Larcker, 1981). The AVE (Average Variance Extracted) value for depression is 0.43, which is less than 0.50; however, the rest are between 0.58 and 0.63, which is greater than 0.5, indicating overall good convergent validity of the model (Hair, 2014). The convergent and discriminant validity of the model are generally in line with requirements.

**Figure 1:**
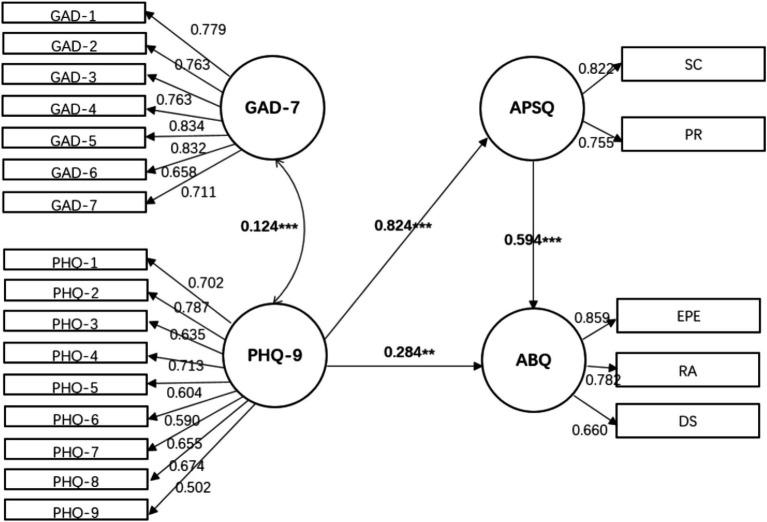
Mediating model of the path between anxiety- > depression- > psychological strain- > athlete burnout. *X*^2^ = 401.14, df = 167, *p* < 0.001, *X*^2^/df = 2.40, SRMR = 0.03, RMSEA = 0.05, CFI = 0.96, GFI = 0.93.

**Figure 2:**
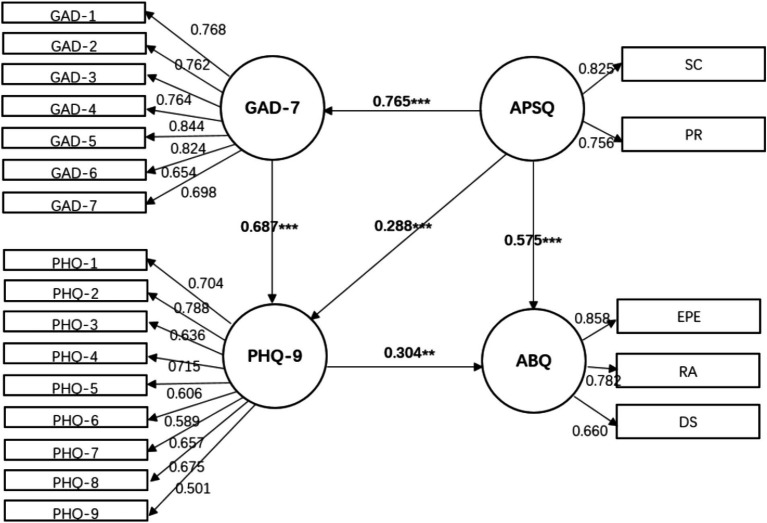
Mediating model of psychological strain- > anxiety- > depression- > athlete burnout. *X*^2^ = 396.37, df = 166, *p* < 0.001, *X*^2^/df = 2.39, SRMR = 0.03, RMSEA = 0.05 CFI = 0.96, GFI = 0.93.

**Figure 3:**
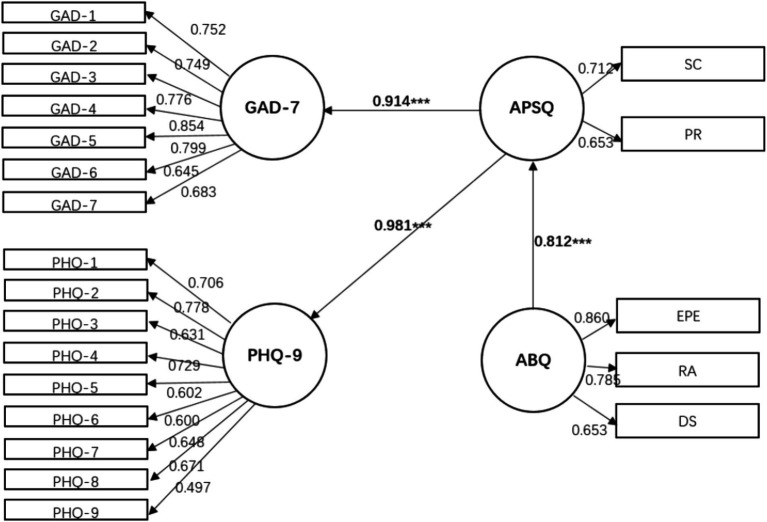
Mediating model of athlete burnout- > psychological strain- > anxiety and depression. *X*^2^ = 450.47, df = 169, *p* < 0.001, *X*^2^/df = 2.67, SRMR = 0.04, RMSEA = 0.06, CFI = 0.95, GFI = 0.92.

Building upon this, an exploratory measurement of the structural relationships among anxiety, depression, psychological pressure, and athlete burnout is conducted, followed by an analysis of the model fit (Figures 1–3).

Model 1: Anxiety and depression are placed at the forefront, directly impacting fatigue, and indirectly influencing it through psychological pressure.

Model 2: Psychological strain is placed in the front, which directly affects athlete burnout, and indirectly affects athlete burnout through anxiety and depression.

Model 3: Athlete burnout is placed in the front, which directly affects psychological strain, and indirectly affects anxiety and depression through psychological strain.

A comparison of the model fits showed that Model 2, where psychological strain measured by the APSQ-8 served as the independent variable and anxiety (GAD-7) and depression (PHQ-9) acted as partial mediators on athlete burnout (ABQ-12), provided a better fit to the data (χ^2^ = 396.37, degrees of freedom = 166, *p* = 0.000, χ^2^/df = 2.39, SRMR = 0.034, RMSEA = 0.05, CFI = 0.96, GFI = 0.93). The path coefficients for the latent variable relationships fell within the range of 0.29–0.76. All three models consistently revealed that the mutual influence between anxiety (GAD-7) and burnout (ABQ-12) was insignificant, while the direct paths from depression (PHQ-9) and psychological strain (APSQ-8) to burnout were statistically significant, and indirect paths were also present in the models.

#### 3.4.1 Mediation effect measurement

Building upon Model 2, the direct and indirect effects of anxiety, depression, and psychological strain on athlete burnout were calculated, as presented in Tables 5, 6.

**Table 5:** List of path coefficients.

Path	Standardized coefficient	Residual variance	Standard error	Critical ratio	*p*
APSQ➔ABQ	0.58	0.83	0.14	5.91	**
APSQ➔GAD-7	0.77	0.14	0.01	11.96	**
APSQ➔PHQ-9	0.29	0.03	0.01	4.59	**
GAD-7➔PHQ-9	0.69	0.41	0.05	8.04	**
PHQ-9➔ABQ	0.30	4.07	1.23	3.31	**

**p<0.01.

**Table 6:** Summary of mediating effects.

Path	Standardized coefficient	Residual variance	*p*	95% Confidence Interval	
				Lower	Upper
APSQ➔PHQ-9➔ABQ	0.09	0.13	0.02*	0.04	0.24
APSQ➔GAD-7➔PHQ-9➔ABQ	0.16	0.23	0.05*	0.01	0.44
Total indirect effect	0.25	0.36	0.05*	0.00	0.59
The difference between the two mediation paths	0.07	0.10	0.04*	0.00	0.24
APSQ➔ABQ//direct effect	0.58	0.83	0.00**	0.52	1.30
Total effect	0.82	1.18	0.00**	1.01	1.37
Proportion of total indirect effects	30.05%				

**p<0.01, *p<0.05.

Bootstrap method (*n* = 2000) was used to analyze the reliability of the influence paths, and 95% confidence intervals of each path were found.

The direct and indirect effect analysis of this model found that the direct effect coefficient of psychological strain (APSQ) on athlete burnout (ABQ) was 0.58, the indirect effect coefficient of psychological strain (APSQ) mediated by anxiety and depression on athlete burnout was 0.25, and the mediating effect of indirect influence path accounted for 30.05%.

## 4 Discussion

### 4.1 Features of athlete mental health indicators

In this study, the overall reported moderate to severe levels of anxiety among athletes was 11.38%, and the reported moderately severe to severe levels of depression was 16.77%, while the reported high to very high level of Athlete Psychological Strain (APSQ) was 78.64%. The detection rate of anxiety was lower than those found in previous research, but the rate of depression was higher in previous research (Zhu et al., 2021). Previous rates for depression in athletes could range from 15.6 to 21% (Proctor and Boan-Lenzo, 2010), and even up to 33.5% (Armstrong and Oomen-Early, 2009). However, the detection rate for psychological strain in this study was significantly higher than in previous studies (29.5, 38, 50.6%; Christensen et al., 2021; Anderson et al., 2023; Yoshida et al., 2023).

There are numerous factors that can affect the mental health of athletes, including gender, age, body image, the type of sport in the training environment, coach-athlete relationships, team culture, and pre-competition stress, among others.

The brain, being a vital organ of the human body, if troubled by psychological issues, can prevent athletes from performing at their best in sports or in life. As a special group, athletes do not differ significantly from the general population in terms of mental health and are not immune to psychological issues such as anxiety and depression. In fact, due to the particularities of their profession, they may face even greater psychological pressures. Some scholars have identified elite young athletes as a high-risk group for mental health issues (Gerber et al., 2022). It is crucial to pay attention to the mental health issues of young athletes, as they can significantly impact their future development and prospects.

### 4.2 Characteristics of athlete burnout

Athletic career burnout is categorized into three dimensions: physical and emotional exhaustion, diminished accomplishment, and devaluation of sports. Physical and emotional fatigue is closely related to intense competition and training, while the reduction in accomplishment is associated with the long-term unmet needs. The severity of these three dimensions progresses sequentially (Gould and Whitley, 2009). The initial state of athlete burnout manifests as emotional issues, such as aversion to training, which then evolves into cognitive problems, such as inattention, and eventually leads to behavioral changes, such as skipping training, declining performance, and withdrawal from sports. Compared to the first three indicators of mental health, there is less research on athlete burnout, and its antecedent, protective, and effect factors remain unclear. In China, the term “sports psychological fatigue” has long been used as a substitute definition for athlete burnout, which to some extent obscures the true psychological issues that the term burnout represents. The term “burnout” implies fatigue and disinterest in an activity (burned out), as well as laziness, slackness, and disrespect (negligent). It reflects an attitude and behavioral intention towards a professional environment. In contrast, “sports psychological fatigue” only implies fatigue without the aspect of negligence, which deviates from and is an incomplete representation of the true definition of burnout.

Athletes engaged in long-term high-intensity sports training will exhibit a transition from fatigue to burnout. This is distinct from the manifestations of anxiety, depression, and perceived psychological strain. Previously, overtraining was considered the primary cause of athlete burnout, and was even equated with the overtraining syndrome (Armstrong and VanHeest, 2002). Physiologically, overtraining leads to changes in the central nervous system, particularly alterations in the hypothalamic–pituitary–adrenal (HPA) axis and the sympathetic-adrenal-medullary (SAM) axis, as well as disruptions in endocrine function (cortisol, catecholamines, and reproductive hormones), and impairments in immune function, all of which are related to burnout. However, there have been no breakthrough findings on how these physiological changes lead to the development of athlete burnout. From a sociological perspective, perfectionism has long been regarded as a major factor in the development of athlete burnout. Athletes, who consistently aim for the goal of “higher, faster, stronger” in training and competition, may experience burnout when their performance does not align with these goals, and when they fear negative evaluation and rejection from others (Hill and Curran, 2016). Motivation has been found to mediate the relationship between perfectionism and burnout (Madigan et al., 2016), with perfectionism preceding athlete burnout and potentially contributing to its development. Additionally, social networks and support have been proven to be negatively correlated with burnout (DeFreese and Smith, 2013; Isoard-Gautheur et al., 2016), which relates to the health behavior change theory at the interpersonal level. Furthermore, it is noteworthy that athletes’ anxiety, depression, and burnout are closely linked to their self-perception and bodily sensations (Werner et al., 2009). Highly self-obsessed individuals are more susceptible to reacting to internal feelings like anxiety. A heightened focus on the self can exacerbate negative effects such as depression and anxiety, leading to increased sensitivity to stress and reduced resilience to problems, all of which can impact self-representation and decision-making abilities in those with high self-awareness (Sebri et al., 2023).

### 4.3 Interactions between mental health indicators and burnout

This study confirms the significant correlations between APSQ, anxiety, depression, and burnout in the Chinese elite athlete population (Hypothesis 1), as cross-sectional data also reveal that APSQ may have a direct impact on burnout, while anxiety and depression partially mediate the effect of APSQ on burnout (Hypothesis 2). The positive correlations between athlete burnout and various dimensions with mental health indicators, in line with previous findings (*r* = 0.402–0.532) (Lin et al., 2022), were found to be relatively stronger in this study (*r* = 0.53–0.66). The highest correlation was observed between the physical/emotional exhaustion dimension and perceived stress, whereas previous research had identified a stronger connection between the reduced sense of accomplishment dimension and perceived stress (Lee et al., 2017). The strong positive association between burnout and depression is widely supported by numerous studies (Glass and McKnight, 1996; Bianchi and Laurent, 2015). A significant positive correlation between burnout and anxiety was also observed, though generally weaker than that with depression (Sun et al., 2012; Koutsimani et al., 2019). Some studies have reported significant positive correlations between burnout, depression, and perceived stress, but not with anxiety (Crudden et al., 2023).

The path analysis in this study revealed a better fit for the model: stress → anxiety → depression → burnout. Athletes progress from perceived stress to burnout through a sequence where stress perception comes first, followed by anxiety and depression, and burnout at the end. This aligns with earlier research that suggests stress as a precursor, depression and burnout occurring concurrently, and a weaker connection between burnout and anxiety. Prior studies considered stress (or stress response) as a precursor to depression and burnout (Pall, 2001; Plieger et al., 2015). At an early stage of stress perception, individuals strive to maintain high performance levels (Edelwich and Brodsky, 1980). As stress coping strategies fail, anxiety arises. If high performance does not yield rewards, individuals may abandon stress coping later (van Dam, 2016). Burnout is not a short-term response to stress but a later stage when stress resources are depleted (Schaufeli and Bakker, 2004). However, more research is needed to address the overlap between burnout and mental health indicators, as burnout may coexist with both anxiety and depression, with depression often indicating a more severe level of burnout (Ahola et al., 2014). A shared biological basis for this co-development might be DNA methylation, a biomarker for mental disorders (Bakusic et al., 2017). Maslach posits that burnout is a manifestation of a specific work environment, while depression often permeates all aspects of life. The fundamental factor connecting burnout and depression is the inability to cope with environmental stress (Maslach et al., 2001). In summary, 70% of the direct impact on burnout from perceived stress is observed, with 30% mediated through anxiety and depression. This suggests that addressing burnout in athletes should first focus on helping them manage stress, while also paying attention to the erosion of mental health due to daily stress-induced anxiety and depression.

The transition from perceiving stress to the emergence of burnout is a complex psychological and behavioral process, and burnout is a relatively overlooked mental health issue among athletes. Future research should emphasize this process and implement early interventions. The International Olympic Committee’s Athlete Mental Health Assessment Tool (SMHAT-1) also prioritizes screening for stress perception, followed by anxiety, depression, sleep, and diet. As burnout theory and tools mature, they may be incorporated into screening tools, deepening the assessment of athlete mental health.

## 5 Limitation

This study has the following limitations: (1) The cross-sectional design restricts the ability to establish a causal relationship between mental health indicators and athlete burnout. Longitudinal studies would better capture time-related changes; (2) The sample is limited to Chinese athletes, which may not be representative of athletes from diverse cultural backgrounds or environments. The focus on a few sports may not generalize to athletes in other, unrepresented disciplines; (3) The study design did not account for potential factors influencing the relationship between mental health and burnout, such as social support (Zhao et al., 2022), training intensity (Poucher et al., 2022), stress management strategies (Cláudia et al., 2010), and individual bodily perception (Sebri et al., 2023). Future research should incorporate these factors to enhance the comprehensiveness of the study.

## 6 Conclusion

This study aimed to examine the characteristics and relationships of anxiety, depression, psychological strain, and burnout in high-level Chinese athletes. The findings revealed similarities with previous studies, but also highlighted unique aspects. The study discovered significant correlations among these variables. The structural equation analysis showed that psychological strain had both direct and indirect effects on burnout, with anxiety and depression acting as mediators. Anxiety did not appear to have a significant direct impact on burnout.

## 7 Implications

For future research on athletes’ mental health, there should be a focus on enhancing the study of tools like the ABQ and standardizing norm data. It is recommended that the IOC’s Sport Mental Health Assessment Tool 1 (SMHAT-1) incorporates burnout as a component. Sports teams should provide the best service model, including education, diagnosis, screening, and treatment (Moesch et al., 2018), with a clear exit mechanism and treatment plan for athletes who meet clinical intervention criteria (Chang et al., 2020), or alternative therapies tailored to the sports environment. Athletes individually need to raise mental health consciousness, seek social support (Wendling et al., 2018), and develop effective coping strategies (Hooper et al., 2022).

## Data availability statement

The original contributions presented in the study are included in the article/Supplementary material, further inquiries can be directed to the corresponding author.

## Ethics statement

Ethical review and approval was not required for the study on human participants in accordance with the local legislation and institutional requirements. Permission was obtained from coaches of high-level sports teams, both managers and athletes, after they signed an informed consent form.

## Author contributions

YG: Conceptualization, Data curation, Investigation, Resources, Supervision, Writing – review & editing. LW: Conceptualization, Formal analysis, Funding acquisition, Methodology, Software, Supervision, Visualization, Writing – original draft.

## Appendix

Introduction of athlete sample characteristics.

TABLE A1 Detailed information of the participants.

**Table:**

Variable	**Total** ***N* (%)**	**International Master Athlete** ***N* (%)**	**Master Athlete** ***N* (%)**	**Division I Athlete** ***N* (%)**
Gender				
Male	256(51.1)	19(3.8)	109(21.8)	128(25.5)
Female	245(48.9)	24(4.8)	92(18.4)	129(25.7)
Age				
18	94(18.8)	2(0.4)	27(5.4)	65(13.0)
19	110(22)	4(0.8)	28(5.6)	78(15.6)
20	81(16.2)	5(1.0)	37(7.4)	39(7.8)
21	49(9.8)	2(0.4)	22(4.4)	25(5.0)
22	69(13.8)	4(0.8)	36(7.2)	29(5.8)
23	26(5.2)	3(0.6)	13(2.6)	10(2.0)
24	18(3.6)	4(0.8)	12(2.4)	2(0.4)
25	22(4.4)	5(1.0)	12(2.4)	5(1.0)
26	16(3.2)	5(1.0)	7(1.4)	4(0.8)
27	8(1.6)	6(1.2)	2(0.4)	/
28	4(0.8)	1(0.2)	3(0.6)	/
29	4(0.8)	2(0.4)	2(0.4)	/
Sport Discipline				
Swimming	78(15.6)	3(0.6)	32(6.4)	43(8.6)
Track and Field	70(14)	10(2.0)	24(4.8)	36(7.2)
Gymnastics	25(5.0)	4(0.8)	10(2.0)	11(2.2)
Rowing	11(2.2)	/	1(0.2)	10(2.0)
Table Tennis	28(5.6)	1(0.2)	7(1.4)	20(4.0)
Badminton	8(1.6)	1(0.2)	3(0.6)	4(0.8)
Basketball	5(1.0)	/	1(0.2)	4(0.8)
Canoeing/Kayaking	8(1.6)	/	4(0.8)	4(0.8)
Cycling	10(2.0)	/	5(1.0)	5(1.0)
Boxing	5(1.0)	2(0.4)	1(0.2)	2(0.4)
Fencing	10(2.0)	/	1(0.2)	9(1.8)
Modern Pentathlon	17(3.4)	1(0.2)	10(2.0)	6(1.2)
Judo	1(0.2)	1(0.2)	/	/
Tennis	45(9.0)	3(0.6)	24(4.8)	18(3.6)
Martial Arts	17(3.4)	1(0.2)	8(1.6)	8(1.6)
Acrobatics gymnastics	33(6.6)	4(0.8)	20(4.0)	9(1.8)
Taekwondo	2(0.4)	1(0.2)	1(0.2)	/
Calisthenics	2(0.4)	/	/	2(0.4)
Trampoline	18(3.6)	/	6(1.2)	12(2.4)
Rugby	7(1.4)	/	3(0.6)	4(0.8)
Soccer	30(6.0)	4(0.8)	12(2.4)	14(2.8)
Dive	71(14.2)	7(1.4)	28(5.6)	36(7.2)
